# Deep Learning for Ultrasonographic Assessment of Temporomandibular Joint Morphology

**DOI:** 10.3390/tomography11030027

**Published:** 2025-02-27

**Authors:** Julia Lasek, Karolina Nurzynska, Adam Piórkowski, Michał Strzelecki, Rafał Obuchowicz

**Affiliations:** 1 Faculty of Geology, Geophysics and Environmental Protection, AGH University of Krakow, 30-059 Krakow, Poland; julial@agh.edu.pl; 2 Department of Algorithmics and Software, Silesian University of Technology, 44-100 Gliwice, Poland; 3 Department of Biocybernetics and Biomedical Engineering, AGH University of Krakow, 30-059 Krakow, Poland; 4 Institute of Electronics, Lodz University of Technology, 93-590 Lodz, Poland; michal.strzelecki@p.lodz.pl; 5 Department of Diagnostic Imaging, Jagiellonian University Medical College, 30-663 Krakow, Poland

**Keywords:** deep learning, temporomandibular joints, ultrasonography, artificial intelligence

## Abstract

Background: Temporomandibular joint (TMJ) disorders are a significant cause of orofacial pain. Artificial intelligence (AI) has been successfully applied to other imaging modalities but remains underexplored in ultrasonographic evaluations of TMJ. Objective: This study aimed to develop and validate an AI-driven method for the automatic and reproducible measurement of TMJ space width from ultrasonographic images. Methods: A total of 142 TMJ ultrasonographic images were segmented into three anatomical components: the mandibular condyle, joint space, and glenoid fossa. State-of-the-art architectures were tested, and the best-performing 2D Residual U-Net was trained and validated against expert annotations. The algorithm for joint space width measurement based on TMJ segmentation was proposed, calculating the vertical distance between the superior-most point of the mandibular condyle and its corresponding point on the glenoid fossa. Results: The segmentation model achieved high performance for the mandibular condyle (Dice: 0.91 ± 0.08) and joint space (Dice: 0.86 ± 0.09), with notably lower performance for the glenoid fossa (Dice: 0.60 ± 0.24), highlighting variability due to its complex geometry. The TMJ space width measurement algorithm demonstrated minimal bias, with a mean difference of 0.08 mm and a mean absolute error of 0.18 mm compared to reference measurements. Conclusions: The model exhibited potential as a reliable tool for clinical use, demonstrating accuracy in TMJ ultrasonographic analysis. This study underscores the ability of AI-driven segmentation and measurement algorithms to bridge existing gaps in ultrasonographic imaging and lays the foundation for broader clinical applications.

## 1. Introduction

Temporomandibular joint (TMJ) disorders are among the most significant causative factors of orofacial pain [1]. According to the latest meta-analysis, approximately 34% of the global population is affected by conditions related to TMJ dysfunction [2]. Geographically, TMJ disorders are most prevalent in South America, potentially due to socio-demographic and genetic factors, though these remain poorly understood [3].

Research consistently indicates that the etiology of TMJ disorders is multifactorial, with individual prevalence varying based on specific contributing factors [4,5,6,7]. Commonly cited causative factors include parafunctional habits, psychological conditions such as depression and anxiety (often accompanied by increased muscle tension), and joint degeneration due to hypermobility, trauma, and inflammation. Additionally, autoimmune diseases, rheumatoid disorders, and fibromyalgia have been linked to prolonged TMJ pain [8,9,10,11,12,13].

Among the morphological and functional parameters used to assess TMJ health, the width of the joint space is particularly critical for determining the status of the orthognathic system [14]. Changes in joint space width, especially reductions, are strongly associated with morphological disorders such as arthrosis and other degenerative TMJ conditions [15,16]. Traditional imaging techniques, such as computed tomography (CT), cone-beam computed tomography (CBCT), and magnetic resonance imaging (MRI), are widely used for evaluating TMJ space, demonstrating high levels of agreement in results [17]. However, these techniques present several limitations in the context of temporomandibular joint (TMJ) assessment. While CT and CBCT are highly effective for evaluating bony structures, they offer limited contrast for soft tissue visualization, which is critical for assessing the articular disk and synovial membrane of the TMJ [18]. Additionally, these modalities rely on ionizing radiation, raising concerns about repeated exposure, particularly for patients with chronic TMJ disorders. CT and CBCT imaging are also prone to artifacts caused by metallic dental restorations, which can compromise image quality and diagnostic accuracy [19]. Furthermore, these techniques usually provide static images, making it difficult to evaluate dynamic TMJ movements that are crucial for understanding joint function. If pseudo-dynamic assessment is held, this technique is associated with multiplied radiation dose and, therefore, is considered not safe [20]. MRI, on the other hand, excels in soft tissue visualization without radiation exposure but is not without its challenges. It is costly, time-intensive, and less accessible compared to other imaging modalities. Motion artifacts during MRI scans can degrade image quality, and its static nature also limits the ability to capture real-time joint dynamics [21,22]. Moreover, MRI is contraindicated for patients with metallic implants, pacemakers, or claustrophobia [23]. These limitations underscore the need for alternative imaging approaches, such as artificial intelligence-enhanced ultrasonography, which offers real-time, non-invasive, cost-effective, and radiation-free imaging, addressing many of the challenges posed by traditional techniques. Ultrasonography has shown potential in clinical settings, achieving similar results and emerging as a candidate for routine use [24]. However, ultrasonography measurements are subject to operator fatigue and bias, leading to concerns about reproducibility. Such measurements often require significant expertise, limiting their reliability when performed by less experienced clinicians [25,26].

Over the past few years, artificial intelligence (AI) has shown significant potential in addressing various challenges associated with medical imaging. It is worth noting that, due to the high variability and noise levels in ultrasound images, the number of AI algorithms supporting the analysis of such images is limited compared to images from other modalities (CT, MRI, X-ray) [27]. Nevertheless, machine learning methods are applied in the analysis of such images. These methods utilize radiomics, including frequently used image texture features [28], to support image-based diagnosis in a diverse range of applications [29,30,31]. Recently, deep learning approaches have also been employed for processing and analyzing such images, e.g., to improve ultrasound image quality [32] or to assist in the diagnostic process [33].

AI has also been effectively utilized in the evaluation of the TMJ. Vinayahalingam et al. [34] demonstrated the potential of deep learning techniques for the automated segmentation of TMJ structures on MRI. In addition to CBCT, deep learning-based segmentation of the TMJ has also been widely explored in MRI [35,36,37,38,39]. A review of studies on TMJ segmentation across different modalities is summarized in Table 1. Choi et al. [40] utilized AI-enhanced analysis of panoramic radiography and joint noise data to improve the diagnosis of degenerative joint diseases. Furthermore, Kazimierczak et al. [41] showcased the impact of AI in optimizing CBCT imaging quality for TMJ evaluation.

Although AI has been successfully applied to the assessment of the temporomandibular joint using various imaging modalities, to our knowledge, there are currently no AI-based solutions for the ultrasound assessment of the TMJ (Table 1), despite its crucial importance in modern medicine [42]. In light of the above, this study aimed to develop and validate an AI-driven method for the automatic and reproducible measurement of TMJ space width using ultrasonographic imaging. Recognizing the growing role of AI in medical imaging, we sought to evaluate the effectiveness of the developed method using deep learning segmentation architectures for this relatively under-explored imaging modality. To achieve this, a dataset comprising 142 ultrasonographic images of the TMJ was curated and annotated by a radiologist, segmenting the images into three anatomically distinct components: the mandibular condyle, the joint space, and the glenoid fossa. In an effort to advance the field, we have made this dataset publicly accessible, enabling further research and validation of AI-based solutions for the ultrasonographic evaluation of TMJ.

## 2. Materials and Methods

Between April 2020 and June 2022, a total of 300 patients undergoing evaluation for TMJ-related pain were referred for ultrasonographic assessment of the temporomandibular joints. The diagnostic ultrasonographic procedures were conducted at the Department of Radiology and Ultragen Medical Clinic, with patients referred from various clinical departments within the Clinical Hospital.

To ensure consistency and reliability of the results, all measurements were performed by a radiologist with a minimum of six years of experience in ultrasonographic examinations, utilizing ultrasonographic technology. The radiologist was directly involved in the joint visualization and measurement process and personally oversaw the examinations. Ethical approval for the study was obtained from the Institutional Ethics Committee of Jagiellonian University (Approval No. 1072.6120.138.17). The study included patients presenting with facial pain localized around the TMJ. These patients were referred for initial ultrasonographic evaluation as part of their diagnostic workup imaging, which is a part of standard evaluation procedure. This study’s dataset has been published and can be retrieved at https://doi.org/10.5281/zenodo.14760859 (accessed on 29 January 2025).

### 2.1. Exclusion Criteria

1. Age Restrictions: Patients under 18 years of age or over 65 years of age were excluded from the study to ensure the study focuses on the adult population with typical TMJ anatomy;
2. Previous TMJ Surgery: Patients with a history of surgical intervention on the TMJ, as such procedures could alter joint morphology and affect ultrasonographic findings;
3. Severe TMJ Deformities: Cases with congenital or severe acquired TMJ deformities, such as ankylosis, which may limit accurate ultrasonographic evaluation;
4. Systemic Conditions: Patients with systemic disorders affecting the TMJ, such as advanced rheumatoid arthritis, severe osteoarthritis, where deformities were too severe;
5. Severe Dental Conditions: Presence of extensive dental malocclusion or recent dental surgeries, which may confound TMJ-associated pain assessment;
6. Inflammatory or Infectious Conditions: Active TMJ infections or severe inflammatory conditions (e.g., abscesses) that may require immediate medical or surgical intervention;
7. Inability to Undergo Ultrasonography: Patients with conditions limiting their ability to comply with proper positioning or imaging protocols;
8. Inadequate Imaging Quality: Exclusion of scans with poor imaging quality;

### 2.2. Examination Protocol

Patients were positioned in a supine, relaxed state with their jaws in habitual occlusion, ensuring that any splints or oral devices were removed prior to the examination. A layer of ultrasound gel was applied to the skin to optimize acoustic coupling. The examination was performed using the GE HealthCare ultrasound system (GE Versana Premier [GE Healthcare, Chicago, IL, USA]) equipped with a shear wave device and an 8–18 MHz hockey stick probe (GE Healthcare, Chicago, IL, USA).

**Figure 1:**
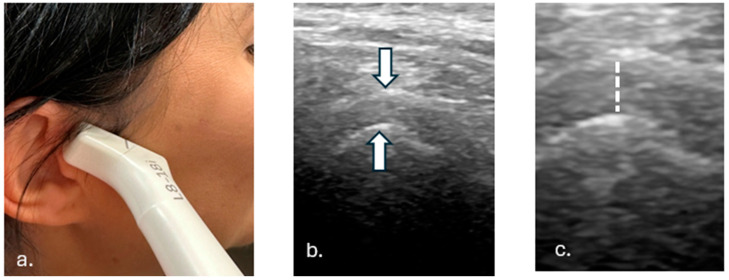
**Figure 1** presents methodology of examination of the TM Joint. (**a**). Hockey stick probe alignment parallel to the joint gap. (**b**). Obtained US image of the joint with indicated articulation surfaces (arrows). (**c**). Joint gap width–searched parameter (marked with dotted line).

The TMJ was evaluated using morphological imaging sections oriented parallel to the joint line. This approach enabled detailed visualization of joint features, including the presence of exudate, irregularities in the bony surface, overall joint congruence (i.e., the alignment of the joint surfaces), and the position of the articular disk. Assessments were conducted during habitual occlusion and dynamic movements of the jaw, such as opening and closing the mouth upon command (Figure 1).

### 2.3. Data

Following the exclusion of unsuitable cases, the final dataset consisted of 142 exams. Each image was preprocessed by being cropped to the diagnostic field of view. The images were saved in 8-bit grayscale format. The dataset was partitioned using the holdout method with a random split into three subsets: a training set containing 86 exams (60%), a validation set consisting of 21 exams (15%), and a test set comprising 35 exams (25%). The validation set was used for hyperparameter tuning and model optimization, while the test set was reserved for final performance evaluation.

### 2.4. Data Annotation

The segmentation process was designed to annotate three anatomically distinct components of the TMJ: the mandibular condyle (MC), representing the inferior portion of the TMJ; the joint space (JS); and the glenoid fossa of the temporal bone (GF), constituting the superior aspect of the TMJ. In the first phase, voxel-wise segmentation of these structures was meticulously performed by a radiologist, creating a reference dataset to serve as the initial ground truth for preliminary U-Net model training. This model was then used to generate the initial AI-generated segmentations.

In the second phase, the radiologist reviewed and refined the initial manual segmentations with guidance from the AI-generated outputs. These refinements were conducted carefully to maintain the anatomical accuracy of the segmented structures while addressing any inconsistencies between the left and right boundaries. Importantly, these adjustments did not alter critical parameters, such as the measurement of the TMJ space width, but were undertaken to enhance the uniformity of the dataset, thereby optimizing its utility as high-quality training data for further model development. This step provided the final segmentations for the final model training.

### 2.5. Model Evaluation

The evaluation of the model was conducted by comparing its predictions with the ground truths provided on the test set. All metrics were computed using Python version 3.11.9. Segmentation performance was quantified using the following metrics:

Dice Coefficient:

(1) $$\mathrm{Dice}=\frac{2\times \left|P\cap G\right|}{\left|P\right|+\left|G\right|}$$

where *P* and *G* represent the predicted and ground truth segmentations, respectively. Dice measures the overlap between the two sets, with a value of 1 indicating perfect agreement.

Precision:

(2) $$\mathrm{Precision}=\frac{\mathrm{TP}}{\mathrm{TP}+\mathrm{FP}}$$

Precision evaluates the fraction of correctly predicted positive pixels among all predicted positives, where *TP* represents true positives and *FP* false positives.

Recall:

(3) $$\mathrm{Recall}=\frac{\mathrm{TP}}{\mathrm{TP}+\mathrm{FN}}$$

Recall (or Sensitivity) measures the fraction of correctly predicted positive pixels among all actual positive pixels, where *FN* represents false negatives.

Volume Similarity (VS):

(4) $$\mathrm{VS}=1-\frac{\left|V_{P}-V_{G}\right|}{V_{P}+V_{G}}$$

where *V_P_* and *V_G_* are the volumes of the predicted and ground truth segmentations, respectively. VS assesses the relative agreement in size between the predicted and actual segmentations, with a value of 1 indicating perfect volumetric match.

Hausdorff Distance (HD):

$$\mathrm{HD}\left(P,G\right)=\max\left\{\underset{p\in P}{\sup}\underset{g\in G}{\inf}d\left(p,g\right),\underset{g\in G}{\sup}\underset{p\in P}{\inf}d\left(g,p\right)\right\}$$

Hausdorff Distance (HD) quantifies the maximum boundary deviation between predicted (*P*) and ground truth (*G*) segmentations, computed as the greatest distance from any point in one set to the closest point in the other.

### 2.6. Postprocessing

Postprocessing was applied to refine the predicted segmentation masks by analyzing connected components for each class (excluding the background). For each class, contiguous regions (connected components) were identified, and their sizes were calculated. The largest component was used as a reference, and smaller components were retained only if their size was at least 60% of the largest component. This thresholding effectively removed spurious regions while preserving meaningful structures. The processed masks were reconstructed by retaining the relevant components for each class. This method improved segmentation quality by reducing noise and focusing on significant structures.

### 2.7. TMJ Space Width Measurement Algorithm

To measure the TMJ space width (JSW), a computational algorithm was developed to identify and calculate the vertical distance between the mandibular condyle and the glenoid fossa in segmented data. The algorithm first locates the superior-most point of the MC, defined as the pixel with the highest vertical position within its region. If multiple pixels share this vertical position, the algorithm selects the median pixel along the horizontal axis. Subsequently, the algorithm identifies the closest point on the GF directly aligned vertically with the selected MC point within the same column. The JSW is then determined by calculating the vertical distance between these two points.

## 3. Results

### 3.1. Architecture Selection

To identify the most effective segmentation architecture, we evaluated multiple state-of-the-art deep learning models, including Residual U-Net [43], U-Net++ [44], SegResNet (with and without Variational Autoencoder) [45], V-Net [46], Attention U-Net [47], and DeepLabV3 [48]. All training and inference experiments were conducted on an NVIDIA RTX 3080 GPU (NVIDIA Corporation, Santa Clara, CA, USA). Each model was trained using an automated hyperparameter tuning approach to optimize performance. Model selection was performed using Ray Tune [49], along with the Asynchronous Successive Halving Algorithm, which dynamically pruned underperforming configurations, enabling computationally efficient exploration of the hyperparameter space. The entire optimization process was systematically tracked and logged using Weights & Biases [50]. The hyperparameter search space was defined to optimize key model components, including optimizer type, learning rate, number of feature channels, activation function, normalization method, kernel size, and dropout rate. The specific parameters varied depending on the architecture to ensure compatibility with the network’s design. The optimal hyperparameters for each architecture were selected based on the mean Dice score on the validation set.

Subsequently, the best-performing architectures were evaluated on the test set using clinically relevant and computational metrics: segmentation accuracy (Dice coefficient, Hausdorff Distance [mm], precision, recall, volume similarity), training efficiency (number of epochs, epoch-wise training time [s]), inference speed (per-image inference latency [s]), and model complexity (number of parameters, multiply–accumulate operations [MACs]). A comprehensive comparison of these architectures is presented in Table 2. Given its demonstrated segmentation accuracy and efficiency, the Residual U-Net was identified as the optimal model for further refinement and final validation.

### 3.2. Optimal Hyperparameter Configuration

**Figure 2:**
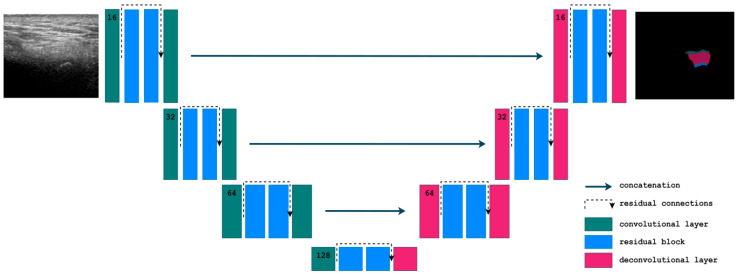
Schematic representation of the Residual U-Net architecture.

The final model was a 2D Residual U-Net implemented within the MONAI framework [51]. This model utilized a single-channel input and a four-channel output, with a network configuration consisting of feature channels [16, 32, 64, 128], with strides of 2 and incorporated 16 residual units per block, instance normalization, PReLU activation, and dropout (0.3) for regularization (Figure 2).

**Figure 3:**
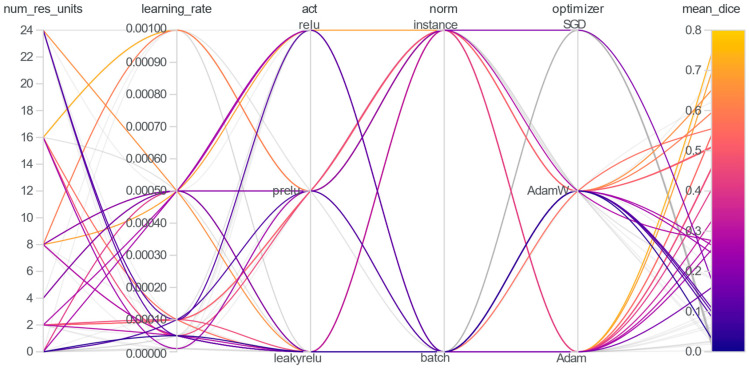
The plot visualizes the performance of different hyperparameter combinations for a subset of experiments.

The model was trained for 150 epochs with a batch size of 2, using employed Dice Loss with softmax normalization and was optimized via the Adam optimizer with a starting learning rate of 1 × 10^−3^, using a ReduceLROnPlateau scheduler all implemented within the PyTorch framework (version 2.5.1). To further improve segmentation performance and model stability, the final model was trained multiple times (with the same hyperparameters), and the three best runs—determined based on the validation Dice score—were selected for ensembling, enhancing robustness, and reducing variance in predictions. Figure 3 provides an overview of a selected subset of explored hyperparameter configurations and their corresponding mean Dice scores on the validation set.

### 3.3. Final Model Segmentation Performance

**Figure 4:**
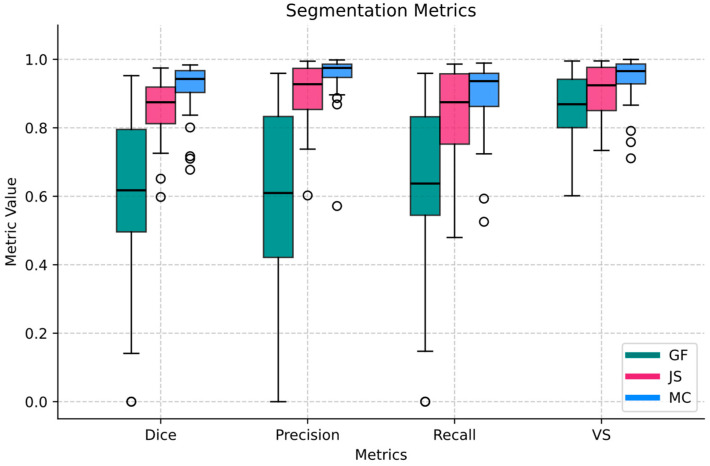
Segmentation performance metrics for the mandibular condyle (MC), joint space (JS), and glenoid fossa (GF) on the test set. Metrics include Dice coefficient, precision, recall, and volume similarity present as boxplots. Circles represent outliers.

The segmentation performance of the model was evaluated across three anatomical structures: the mandibular condyle, the joint space, and the glenoid fossa of the temporal bone. The results, summarized in Table 3 and Figure 4, present the Dice coefficient, precision, recall, and volume similarity (VS) for each structure.

The MC achieved the highest performance across all metrics, with a Dice coefficient of 0.91 ± 0.08, precision of 0.95 ± 0.07, recall of 0.89 ± 0.11, and VS of 0.94 ± 0.07. These values indicate highly accurate segmentation with minimal volume discrepancies. The joint space (JS) also demonstrated strong segmentation performance, with a Dice coefficient of 0.86 ± 0.09, precision of 0.90 ± 0.09, recall of 0.84 ± 0.15, and VS of 0.90 ± 0.08. While the high precision suggests accurate predictions, the slightly lower recall indicates occasional under-segmentation. In contrast, the GF exhibited the lowest performance, with a Dice coefficient of 0.60 ± 0.24, precision of 0.60 ± 0.27, recall of 0.63 ± 0.25, and VS of 0.86 ± 0.10. The larger variability in these metrics highlights challenges in segmenting this structure, potentially due to its intricate geometry and less-defined boundaries.

Overall, the model demonstrated robust segmentation for the mandibular condyle and joint space, albeit with greater variability in performance for the glenoid fossa.

### 3.4. TMJ Space Width Measurement

The algorithm for measuring TMJ space width was evaluated on the same test set used to assess the segmentation model. Measurements were derived from manual segmentations (referred to as reference measurements), which were performed by a radiologist to serve as the ground truth, and AI model predictions (referred to as AI measurements) to assess the model’s performance in comparison to expert-defined standards. The mean TMJ space width obtained from the AI predictions was 1.80 mm, with a median of 1.67 mm. In comparison, the reference measurements yielded a mean of 1.88 mm, with the same median of 1.67 mm. The mean difference between the AI and reference measurements was 0.077 mm, indicating minimal systematic bias. The standard deviation of the differences was 0.33 mm, reflecting the variability between the two sets of measurements.

The mean percentage error was 8.96%, and the mean absolute error was 0.18 mm. These results suggest that the algorithm performs consistently and accurately. Such findings highlight the algorithm’s potential for reliable and automated quantification of TMJ space width in clinical and research applications. A statistical summary of the results is provided in Table 4.

**Figure 5:**
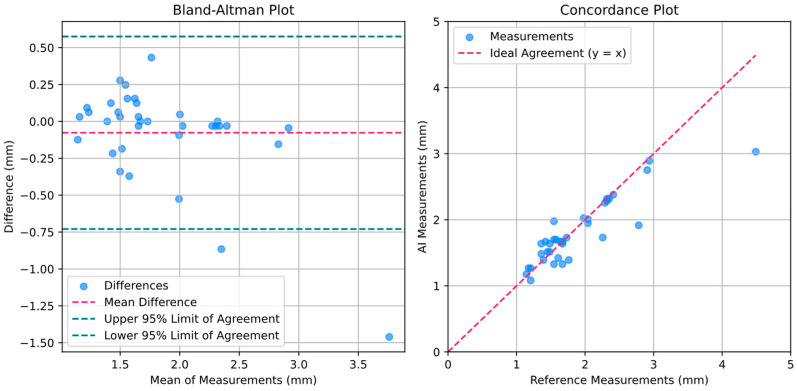
Bland–Altman and concordance plots for assessing the agreement between AI-predicted TMJ space width measurements and reference measurements.

The quality of the automatic measurements was further assessed through the graphs in Figure 5, which include the Bland–Altman plot and the concordance plot. The Bland–Altman plot evaluated the agreement between the AI-predicted TMJ space width measurements and the reference measurements obtained from manual segmentations. The x-axis represents the mean of the AI and reference measurements, while the y-axis shows the difference between the two measurements. Individual data points, displayed in blue, represent the differences across the test set. The dashed red line denotes the mean difference, highlighting any systematic bias, while the dashed green lines represent the upper and lower 95% limits of agreement. Notably, the mean difference lies close to zero, reflecting minimal systematic bias between the AI predictions and the reference standard. Furthermore, nearly all measurements (except two) fall within the limits of agreement, underscoring the consistency of the AI model. The two outliers were both underestimations, which may be attributed to the possibility that the AI model incorrectly segmented the glenoid fossa, potentially marking a different anatomical structure or delineating the region much too broadly. It is worth noting that one of the outliers corresponded to an exceptionally large reference measurement compared to the overall dataset, which could indicate that the deviation was influenced by the specific characteristics of this particular measurement. This suggests that the issue may be a result of the limited diversity within the dataset. In a more diverse and representative dataset, such extreme cases might be less frequent or entirely absent, thereby reducing the overall impact of outliers on the evaluation of the model’s performance.

The concordance plot assessed the agreement between the AI-predicted measurements and the reference measurements. The x-axis corresponds to the reference measurements, and the y-axis corresponds to the AI-predicted measurements. Individual data points are displayed in blue, while the dashed red line represents the line of perfect agreement. The alignment of data points along this line demonstrates a high degree of agreement between the two measurement sets.

Both graphs underscore the high concordance and accuracy of the AI model in predicting TMJ space width when compared to the reference standard, confirming the robustness and reliability of the proposed algorithm.

**Figure 6:**
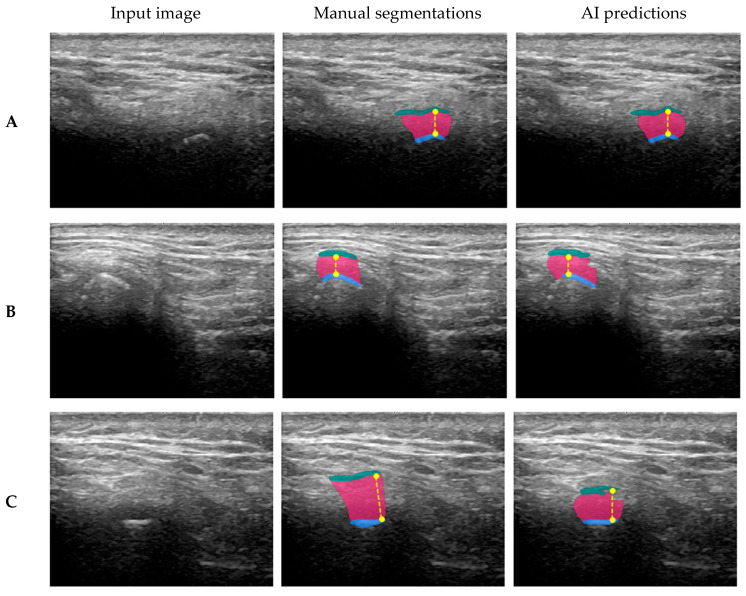
The visualization showcases TMJ segmentation and joint space width (yellow dashed line) measurement results, highlighting the mandibular condyle (MC), joint space (JS), and glenoid fossa (GF) with distinct colors: MC in blue, JS in pink, and GF in green. Segmentation performance is demonstrated across three scenarios: (**A**) the highest Dice (GF: 0.89, JS: 0.96, MC: 0.97), (**B**) the average Dice (GF: 0.78, JS: 0.91, MC: 0.84), (**C**) and the lowest Dice (GF: 0.00, JS: 0.65, MC: 0.84).

The results of both the AI model for TMJ segmentation and the TMJ space width measurement are visually presented in Figure 6, highlighting performance across varying levels of accuracy. The figure includes representative cases from the test set, categorized by best, average, and worst Dice scores. Specifically, segmentation performance is illustrated for the highest Dice scores ((A) GF: 0.89, JS: 0.96, MC: 0.97), the average Dice scores ((B) GF: 0.78, JS: 0.91, MC: 0.84), and the lowest Dice scores ((C) GF: 0.00, JS: 0.65, MC: 0.84). The mandibular condyle is segmented with high accuracy, highlighting the model’s reliability in identifying this anatomical structure. Deviations in TMJ space width measurements are primarily attributable to under-segmentation of the glenoid fossa, as illustrated in the example study (C). Occasionally, the model may incorrectly segment a different structure due to the challenges of interpreting ultrasonographic signals. One potential source of segmentation error is the influence of surrounding ligaments, which can sometimes appear as hyperechoic (bright) structures on an ultrasound, potentially leading to misclassification. Despite these occasional inaccuracies, the model demonstrates strong overall performance. With further refinements and the incorporation of additional training data, the AI model holds significant potential as a valuable tool for automating TMJ assessments.

## 4. Discussion

Among the various radiological modalities used for assessing the TMJ, ultrasonography (US) has gained popularity among imaging professionals due to its affordability, ease of use, and non-invasive nature [52]. However, concerns remain regarding its accuracy, as the imaging setup can be difficult to replicate. Variations in patient anatomy and the subjective approach of the examiner necessitate standardization to improve reliability [53,54]. In this study, the mean distance between the TMJ articulating surfaces measured using ultrasound (US) was 1.9 mm. This finding does not align with the results reported by Noh et al. [26], who observed a mean distance of 3 mm in their studied population. This discrepancy may be attributed to the inclusion of patients with varying conditions in our study. Specifically, cases with disk displacement and mild arthrosis were included to develop a more robust system capable of handling diverse datasets. While artificial intelligence (AI) has been employed in various studies for detecting and differentiating pathologies, its application in TMJ ultrasonography remains unexplored. Lee et al. [55] utilized convolutional neural networks (CNNs) to automatically detect effusion in the TMJ, diagnosed via MRI, achieving a specificity of 87.25% with their proposed model. Similarly, Xu et al. [56] reviewed CNN-based detection of osteoarthrosis in X-ray images, reporting diagnostic accuracies ranging from 78.0% to 92.4%, highlighting the potential of AI as a promising diagnostic tool. In a study by Almășan et al. [57], artificial neural networks (ANNs) and CNNs were used to detect TMJ osteoarthritis from cone-beam computed tomography (CBCT) images, achieving sensitivity and specificity values of up to 98% and 83%, respectively. Eşer et al. [58] applied the YOLO approach to CBCT image analysis, reporting a sensitivity of 1, further demonstrating the potential of this method. Farook and Dudley [59] reviewed the overall performance of AI techniques based on radiographs and highlighted their strong diagnostic accuracy compared to MRI, particularly for edge detection in X-ray images. Zou et al. [60] used a deep learning model (ANN) to evaluate clinical features, medical status, and psychosocial conditions, comparing the results with those of physicians with varying levels of experience. Their study revealed that the ANN achieved an accuracy of over 90%, outperforming less experienced physicians. Additionally, Yıldız et al. [61] applied machine learning algorithms to evaluate non-radiographic clinical parameters, achieving an accuracy of 86% in their study. The comparative analysis of existing studies underscores the significant advancements in AI applications for CBCT, X-ray, MRI, and even clinical data. However, there remains a notable gap in the application of AI for ultrasonographic analysis of the TMJ. Various methods, predominantly CNNs, have been employed for segmentation and diagnosis in other imaging modalities [34]. Addressing this gap is crucial and could provide valuable insights into the field.

This work represents a step toward standardization in TMJ imaging by utilizing an AI training set based on the expertise of highly experienced physicians. The proposed AI solution has the potential to serve as a reliable second opinion for a broader cohort of medical professionals, offering a significant contribution to the field of TMJ diagnostics and filling a critical gap in current knowledge.

Seven deep neural network architectures designed for image segmentation were compared in this study. Residual U-Net achieved the best segmentation results, as indicated by the Dice, HD, precision, recall, and VS metrics presented in Table 2. This network is based on the classic U-Net architecture but incorporates residual blocks, a solution commonly used in residual networks. These blocks help to mitigate the vanishing gradient problem, which hinders training, especially in networks with a large number of deep layers. A residual block includes a skip connection that adds the original input to the output of the convolutional block, providing an alternative pathway for the gradient. Compared to other architectures, Residual U-Net also exhibited a relatively short inference time (0.23 ms), a moderate number of model parameters (35.3 × 10^6^), and a computational complexity of 3.15 × 10^9^ MACs. The beneficial impact of residual blocks on segmentation accuracy is further supported by the good results obtained with SegResNet and SegResNet VAE, where most quality metrics were only slightly worse than those of Residual U-Net (with the exception of HD, which was notably higher). Additionally, these networks exhibited the shortest inference time (0.09 ms), and SegResNet VAE had the lowest computational complexity, equal to 0.88 × 10^9^ MACs.

While this study utilized 142 ultrasonographic examinations, the relatively small dataset poses limitations to the generalizability of the findings. The lack of diversity in demographic and clinical characteristics may hinder the applicability of the AI model to broader patient populations with varying anatomical or pathological profiles. Future studies with larger, more diverse datasets are essential to validate the robustness and adaptability of the proposed model. Such datasets should include a wide range of ethnicities, age groups, and TMJ conditions to ensure broader clinical applicability. A study on a larger and more diverse dataset to validate the robustness of the AI model is planned as a future work.

The lower performance of the segmentation model for the glenoid fossa (Dice: 0.60 ± 0.24) highlights challenges related to the complex geometry and less-defined boundaries of this structure. This variability could reduce the reliability of joint space width measurements in cases with atypical anatomical features. The inclusion of patients with conditions like disk displacement and mild arthrosis aimed to create a robust system but introduced significant variability in the dataset. This might have influenced model training and performance, potentially limiting its application in studies focused on specific TMJ disorders.

Another critical limitation is the reliance on manual annotations from a single, albeit experienced, expert radiologist. While this ensured high-quality annotations, it introduced the possibility of human bias in defining ground truth, which could have influenced model training and evaluation. To address this, future studies could explore using annotations from multiple experts, complemented by consensus strategies or automated refinement tools, to minimize bias and improve annotation reliability.

## 5. Conclusions

The present AI method for TMJ ultrasonographic assessment has significant clinical implications, particularly in addressing the challenges of variability and reproducibility in TMJ imaging. The developed AI model demonstrates high accuracy in segmenting critical anatomical structures, such as the mandibular condyle and joint space, and provides reliable measurements of TMJ space width with minimal bias compared to manual assessments. This capability is important for standardizing ultrasonographic evaluations, which are traditionally prone to operator fatigue and variability, thereby ensuring consistent and objective results across different clinical settings.

By automating the segmentation and measurement processes, the AI system has the potential to reduce the time and expertise required for TMJ ultrasonographic analysis. This is especially valuable in resource-limited settings where access to highly trained radiologists may be restricted. Moreover, the absence of ionizing radiation and the real-time capabilities of ultrasonography make this AI-driven approach a safer and more practical option for routine TMJ evaluations, particularly for monitoring chronic conditions that require repeated imaging.

Furthermore, its ability to streamline the imaging workflow and offer standardized results could enable broader adoption of ultrasonography as a routine diagnostic tool for TMJ disorders, bridging the gap between high-cost imaging modalities like MRI and CT and the need for accessible, non-invasive diagnostics in everyday practice. This positions the AI method as a transformative tool in the field of TMJ imaging, paving the way for more efficient, and patient-centered care.

## Figures and Tables

**Table 1:** Summary of deep learning studies on TMJ segmentation.

Author(s)	Modality	Segmented Structures
Vinayahalingam et al. (2023) [34]	CBCT	Mandibular condyle, glenoid fossa
Kumar & Baskaran (2023) [35]	X-ray	Mandibular condyle, glenoid fossa
Ito et al. (2022) [36]	MRI	Articular disk
Choi et al. (2024) [37]	CBCT	Mandibular condyle
Li et al. (2022) [38]	MRI	Mandibular condyle, articular eminence, articular disk
Yoon et al. (2024) [39]	MRI	Mandibular condyle, temporal bone, articular disk
Our Study	US	Mandibular condyle, joint space, glenoid fossa

**Table 2:** Summary of evaluated segmentation architectures, including segmentation accuracy, training efficiency, inference speed, and model complexity.

	Attention U-Net	U-Net ++	DeepLabv3	SegResNet	SegResNetVAE	Residual U-Net	V-Net
Dice	0.64	0.72	0.72	0.73	0.73	0.75	0.66
Hausdorff Distance [mm]	5.36	2.14	2.08	2.52	2.76	1.42	3.10
Precision	0.67	0.74	0.75	0.73	0.73	0.77	0.60
Recall	0.65	0.72	0.72	0.74	0.74	0.75	0.79
Volume Similarity	0.87	0.90	0.88	0.90	0.91	0.91	0.79
Number of Epochs	72	76	35	38	73	59	22
Epoch Training Time [s]	25.24	89.72	163.22	22.85	26.25	15.39	42.38
Inference Time [ms]	0.09	0.18	0.45	0.09	0.09	0.23	0.10
Parameters (×10^6^)	31.83	133.78	281.4	11.61	16.64	35.31	110.95
MACs (×10^9^)	1.01	2.41	55.8	24.25	0.88	3.15	9.36

**Table 3:** Segmentation performance metrics for the mandibular condyle (MC), joint space (JS), and glenoid fossa (GF) on the test set. Metrics are reported as mean ± standard deviation.

Metric	GF	JS	MC
Dice	0.60 ± 0.24	0.86 ± 0.09	0.91 ± 0.08
Precision	0.60 ± 0.27	0.90 ± 0.09	0.95 ± 0.07
Recall	0.63 ± 0.25	0.84 ± 0.15	0.89 ± 0.11
VS	0.86 ± 0.10	0.90 ± 0.08	0.94 ± 0.07

**Table 4:** Summary of the key metrics for TMJ space width measurements. The table includes the mean and median values for measurements derived from AI predictions and manual segmentations, along with statistical comparisons.

Metric of TMJ Space Width	Value
AI Predicted Mean (mm)	1.80
AI Predicted Median (mm)	1.67
Reference Mean (mm)	1.88
Reference Median (mm)	1.67
Mean Difference (mm)	0.08
Standard Deviation of Differences (mm)	0.33
Mean Percentage Error (%)	8.96
Mean Absolute Error (MAE, mm)	0.18
Root Mean Square Error (RMSE, mm)	0.34

## Data Availability

The dataset is available at https://doi.org/10.5281/zenodo.14760859 (accessed on 29 January 2025).

## Abbreviations

The following abbreviations are used in this manuscript:

TMJ	temporomandibular joint
US	ultrasonography
CBCT	cone-beam computed tomography
MC	mandibular condyle
JS	joint space
GF	glenoid fossa
